# The role of tumor neogenesis pipelines in tumor progression and their therapeutic potential

**DOI:** 10.1002/cam4.4979

**Published:** 2022-07-13

**Authors:** Zhanqi Fu, Yuan Yuan

**Affiliations:** ^1^ Tumor Etiology and Screening Department of Cancer Institute and General Surgery The First Hospital of China Medical University Shenyang China; ^2^ Key Laboratory of Cancer Etiology and Prevention in Liaoning Education Department The First Hospital of China Medical University Shenyang China; ^3^ Key Laboratory of GI Cancer Etiology and Prevention in Liaoning Province The First Hospital of China Medical University Shenyang China

**Keywords:** neogenesis pipeline, therapeutic target, tumor, tumor invasion, tumor progression

## Abstract

Pipeline formation between tumor cells and the tumor microenvironment (TME) is a key event leading to tumor progression. These pipelines include blood vessels, lymphatics, and membranous vessels (the former two can be collectively referred to as vasculature). Pipeline regeneration is a feature of all solid tumors; it delivers nutrients to tumors and promotes tumor invasion and metastasis such that cancer cells grow rapidly, escape unfavorable TME, spread to secondary sites, generate tumor drug resistance, and promote postoperative tumor recurrence. Novel tumor therapy strategies must exploit the molecular mechanisms underpinning these pipelines to facilitate more targeted drug therapies. In this review, pipeline generation, influencing factors, pipeline functions during tumor progression, and pipeline potential as drug targets are systematically summarized.

## Graphical abstract

Pipeline formation between tumor cells and the tumor microenvironment (TME) is a key event leading to tumor progression. These pipelines include blood vessels, lymphatics, and membranous vessels (the former two can be collectively referred to as vasculature). Pipeline delivers nutrients to tumors, promotes tumor invasion and metastasis, generate tumor drug resistance and promote postoperative tumor recurrence.

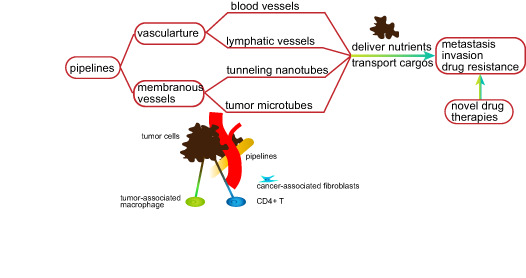

## 1 INTRODUCTION

Pipeline formation between tumor cells and tumor microenvironment (TME) is a key event during tumor progression. These pipelines include blood vessels, lymphatics, and membranous vessels (the former two comprise vasculature). These newly formed pipelines possess relatively unique morphological and structurally unstable characteristics which permit the passage and migration of tumor cells to other sites. Pipeline regeneration is a ubiquitous feature of solid tumors, where tumor cells adaptively respond to TME. As is well‐known, blood and lymphatic vessels are classic pipelines. In the TME, a variety of inflammatory cells participate in the production of paracrine factors, which induce endothelial cells to produce blood vessels and lymphatic vessels,^1^, ^2^, ^3^ or cause tumor cells to imitate endothelial cells to establish new channels providing tumors nutrition and oxygen supplies.^4^ While intercellular membrane tubes that serve as novel pipelines are long‐distance cell‐to‐cell communication means, their structure allows the transportation of multiple cargos between non‐adjacent, non‐homogeneous cells including organelles, hereditary substances, irons, and vesicles.^5^ The existence of tunneling nanotubes (TNTs) greatly accelerated the progression of tumorigenesis, increases tumor invasiveness in tumors, and is the main factor in tumor tissue invasion. Tumor microtubes (TMs), recently observed in gliomas, can redistribute cellular calcium levels.^6^ To date, the mechanisms that underpin pipelines are not fully understood.

Pipeline's substantial contribution to tumor development has drawn increasing attention from researchers  as cancer prognostics are not only affected by the primary tumors but are also related to tumor invasion and metastasis,^7^ where blood and lymphatic vessels can undoubtedly contribute. Meanwhile, TNTs contribute to chemotherapy resistance as they redistribute chemotherapeutic agents, whereas TMs contribute to resistance to surgical lesions.^6^, ^8^ These are issues to be solved in the current research.

New tumor therapy strategies must exploit neogenesis pipelines and use underlying molecular mechanisms to identify new promising drug therapies. In this review, tumor pipeline generation, influencing factors, pipeline functions during tumor progression, and their potential as drug targets are reviewed and summarized.

## 2 CLASSIFICATION AND MORPHOLOGICAL CHARACTERISTICS OF TUMOR NEOGENESIS PIPELINES

The nascent network of pipelines in tumors has a significant difference compared with normal tissues. They have multiple morphologies that are adapted to the TME. Based on their structural composition, pipelines are divided into three categories.

### 2.1 Tumor neovascularization

Tumor neovascularization includes sprouting angiogenesis (SA), intussusceptive angiogenesis (IA, aka vessel splitting or non‐SA), vasculogenic mimicry (VM), and vascular co‐option. Pathological blood vessels formed by tumor neovascularization are distinct to normal blood vessels, they are marked by an irregular vascular network which endothelial cells cannot bind to, they protrude into the vascular cavity, and have no pericyte coverage. The basal lamina is thinner and contains many pores when compared with normal blood vessels, leading to increased permeability and cancer cell infiltration.^9^, ^10^ The disorganized vascular network, with numerous blind‐ended vessels, may cause blood stasis or even reflux.^11^

### 2.2 Tumor lymphangiogenesis

Lymphangiogenesis occurs in the tumor and the tumor edge and constitutes a layer of lymphatic endothelial cells (LECs) measuring 10–60 μm in diameter. Unlike capillaries, lymphatic vessels (LVs) have incomplete discontinuous substrates, or without substrates, and lack smooth muscle cells and pericytes.^12^ LVs in tumor are patchy and chaotic, with an uneven distribution in the tumor, but are usually increased at tumor peripheries. However, in some tumors such as colon cancer patients with metastasis, lymphatic vessel density (LVD) around the tumor is significantly reduced.^13^

### 2.3 Tumor membrane tubes

Membrane tubes are intercellular membrane protrusions, with major cell‐to‐cell communication roles in tumor cells, and structurally facilitate cargo transportation between non‐adjacent, non‐homogeneous cells.^5^ The tubes were first observed to transport mitochondria and viruses and later, bacteria, proteins, lipids, lysosomes, and hereditary material. The framework is mainly composed of actin.^5^, ^14^ Using material transport, damaged cells can be protected from chemotherapy or radiotherapy, thereby increasing tumor progression and invasion. Such membrane tubes are typically classified as TNTs and TMs. TNTs are remote channels consisting mainly of F‐actin which connects different cell types first identified in PC12 (rat pheochromocytoma cell line),^5^ TNTs are now found in several malignancies, for example, bladder cancer^15^ and pleural mesothelioma.^16^ The main morphological features distinguishing TNTs from other actin‐formed structures are intercellular cytoplasmic connections, small diameter, and non‐adhesion to the matrix in vitro culture.^5^ TNTs are not open tubes between two cells, but rather structural adhesions between a cell extension and a cell body.^17^ TMs are primarily located in astrocytic brain tumors, including glioblastomas.^18^ The main difference is that TMs have longer and thicker film extensions and longer lifetimes than TNTs, for example, TNTs’ length is generally in the tens of microns, whereas TMs can reach hundreds of microns. As is typical of most TNTs, in which microtubules are rich in actin, TMs involved more connexin 43.^18^

## 3 FACTORS INFLUENCING NEOGENESIS PIPELINE FORMATION

### 3.1 Angiogenesis and its influencing factors

Tumor angiogenesis or vasculogenesis is a hallmark characteristic of most tumors. The most typical angiogenesis is SA which is a process whereby endothelial cells recombine into new tubular structures, that is, they form new branches from existing blood vessels. IA involves microvessel dilation and vessel wall invagination into the lumen to form an endoluminal column, which eventually leads to vascular division, but usually forms later than SA.^15^ VM was identified in melanoma in 1999^19^ and is a channel formed by tumor cells instead of endothelial cells.^4^ After tumor cells acquire endothelial phenotypes, they spontaneously form conduits to facilitate red blood cell transport. Vascular co‐option is another modality which does not lead to new tube formation but allows tumor cells to move along existing blood vessels and “invade” them. This form of angiogenesis is often observed during glioblastoma.^20^

The factors affecting tumor angiogenesis mainly include TME alterations and a series of regulatory factors induced by rapid tumor growth. Hypoxia is a ubiquitous feature of solid cancers and is caused by a mismatch between cellular oxygen supply and consumption. Hypoxia‐inducible factor (HIF) keeps a major stimulator of tumor angiogenesis, enabling cancer cells to escape unfavorable TME and spread to secondary sites, where HIF exerts key functions. HIF is highly expressed in tumors and directly activates vascular endothelial growth factor (VEGF) and VEGF receptor transcriptome,^21^ which is because HIF, as the main direct regulator of the non‐coding and coding transcriptome, acts by releasing promoter‐paused RNApol2.^22^ For instance, HIF‐2 indirectly increases angiogenic factor expression by regulating long non‐coding RNA (lncRNA).^23^, ^24^ HIF‐1 loss severely retards tumor growth and blood vessel formation.^25^ In hypoxic environments, RKO human colon carcinoma cells adaptively respond; they reduce mitochondria oxygen consumption and facilitate cells’ survival in the reduced reactive oxygen species milieu by trans‐activation of pyruvate dehydrogenase kinase1 (PDK1) and BNIP3 via HIF‐1.^26^ In addition, hypoxia induces epithelial–mesenchymal transition (EMT), which regulates non‐coding RNA in a variety of tumors.^27^ These HIF‐regulated non‐coding RNA molecules are partly derived from exosomes, as exosomes and microvesicles transmit genetic material, like mRNA and mRNA transcripts, between TME^28^. The endothelial uptake of tumor‐derived exosomes activates or reprograms angiogenic signaling pathways^29^ and induces EMT to promote neovascularization.^30^ Moreover, HIFs could modulate multiple signaling pathways that drive EMT. TWIST1, a transcriptional repressor of E‐cadherin in EMT, is also known as the canonical HIF‐induced gene. When HIF‐1α and/or HIF‐2α are deficient, induction of TWIST1 is impaired even under hypoxia.^31^ A typical pathway is a SNAIL1‐SMAD3/4 transcriptional complex that can be activated by transforming growth factor‐β (TGF‐β1) and induces EMT.^32^ While HIF‐1α is responsible for increasing reactive oxygen species via TGF‐β1‐induced metabolic reprogramming and indirectly attenuates the expression of E‐cadherin, HIF‐1α knockdown was also confirmed to reverse the increase in N‐cadherin which is induced by TGF‐β1.^33^ These reports indicated that targeting HIF holds the promise to effectively tackle tumor angiogenesis.

To sustain rapid tumor growth, tumors have to stimulate new blood vessels to facilitate self‐development. This is achieved by the secretion of high levels of angiogenic factors from both tumors and stromal cells, thereby creating active vascular environments.^34^ Many cell types participate in these tumor vasoactive environments. First and foremost, the involvement of tumor‐associated macrophages (TAMs) in tumor angiogenesis is by no means restricted to angiogenic factor release. Macrophages mainly secrete a variety of cytokines directly acting on angiogenesis or indirectly induce angiogenesis after cytokines acting on VEGF. For instance, TAMs directly stimulate neovascularization by secreting the interleukin‐Ilα (IL‐1α) in prostate tumors and by promoting IL‐8 and VEGF production in melanoma. In gliomas, TAMs have been shown to promote VM production via COX‐2 and IL‐6 expression.^35^, ^36^ Metabolic changes of TAMs could also make a difference in angiogenesis. TAMs within the hypoxic niches display significant alterations in metabolism‐related genes like REDD1, an mTOR complex inhibitor. REDD1 knockout TAMs focus more on glycolysis, leading to a competition for glucose with their neighbors and a failure to promote angiogenesis.^37^ Further, cancer‐associated fibroblasts (CAFs) directly facilitate tumor angiogenesis via pro‐angiogenic factors and produce VEGFA. In addition, CAFs secrete chemokine CXCL12, fibroblast growth factor 2, and platelet‐derived growth factor C.^2^ CAFs also promote tumor angiogenesis by influencing the expression of several cytokine‐related genes in breast cancer cells^38^ and indirectly promoting tumor angiogenesis by generating extracellular matrix. WNT2 is mainly produced by CAFs^39^; its expression in the matrix leads to the autocrine activation of typical Wnt signals in endothelial cells. In a xenotransplantation study, subcutaneous tumor growth rates in mice overexpressing WNT2 were increased and tumor vascular density significantly increased, thereby improving tumor angiogenesis.^1^ Additionally, CD4+ T cells such as Th2 and Th17 regulate and enhance angiogenesis and restore perfusion in vivo as they directly enhance endothelial cell migration and germination.^40^ Th2 cells promote blood vessel formation by secreting various cytokines, such as IL‐13, which inhibit blood vessel formation via the JAK2‐STAT6 pathway in vitro.^41^ IL‐17 paracrine network mediates tumor resistance to VEGF and upregulates the pro‐angiogenic cytokines of fibroblasts.^42^ In a recent study, the Th17/IL‐17 axis was dose‐dependently inhibited by IL‐35 and led to VEGF and its receptors’ expression.^43^ IL‐22 promotes the proliferation, migration, and survival of endothelial cells and also promotes the activation of STAT3 in endothelial cells without mediating resistance to anti‐VEGF treatment.^44^

### 3.2 Lymphangiogenesis and its influencing factors

There are three main mechanisms involved in lymphangiogenesis. The first important mechanism involves the proliferation and migration of the endothelial cells into existing lymphatic vessels. When macrophages came into contact with LECs in OCUM‐12 gastric cells, LECs begin to gradually elongate and formed capillary structures, lumen structure expanded,^45^ and new lymphatic vessels were formed. The second mechanism involves the trans‐differentiation of existing vascular endothelial cells; this observation was consistent with evidence suggesting that lymphangiogenesis usually occurred after angiogenesis and requires multiple angiogenic factors. The third mechanism involves a small part of lymphatic vessels that comprise bone marrow‐derived cells (BMDCs). BDMCs differentiate into endothelial progenitor cells and subsequently differentiate into myeloid LECs in the presence of breast cancer patient plasma. These myeloid LECs could induce abundant sprouting of LECs and promote lymphangiogenesis.^46^

VEGF family members include VEGF‐A, VEGF‐B, VEGF‐C, VEGF‐D, and placenta growth factors. Among these, VEGF‐A, ‐C, and ‐D are essential for the sprouting of lymphatic vessels and exert a positive regulatory effect on lymphangiogenesis.^47^ It was shown that inhibited VEGF‐A significantly inhibited tumor lymphangiogenesis and the growth of lymphatic and lung metastasis in the growth of primary breast tumors.^48^ It was also shown that VEGF‐A upregulation was related to angiopoietin‐2 (Ang2) upregulation. Ang2 is an inflammatory mediator with pro‐lymphangiogenic activity, which directly activates tyrosine kinase containing immunoglobulin‐like and EGF‐like domains 2 (Tie2) and also indirectly increases VEGFR‐3 expression.^48^ The VEGF‐C/VEGF‐D/VEGFR‐3 axis is the most important lymphangiogenesis process in tumors. VEGF‐C induces lymphatic vessel sprouting and dilation of draining lymphatic vessels. The blocking of this signaling inhibits LEC proliferation and prevents tumor cells from entering lymphatic vessels.^49^ Thus, it is illustrated that VEGF is the central driving force of lymphangiogenesis.

Additionally, chronic inflammation also contributes to LVD increase. More tumor lymphatic vessels formed when cholangiocarcinoma cells were co‐cultured with lipopolysaccharide‐treated LECs. CXCL2R‐CXCL5 signaling pathway performed a critical role by increasing the expression of EMT determinants corroborating that CXCL5 can induce lymphangiogenesis indirectly on inflamed LECs.^50^ Other inflammatory mediators also play a part in lymphangiogenesis, like TNF‐α and IL‐1β.^51^ The accumulation of M2 TAMs stimulates VEGF‐A and ‐C secretion and promotes lymphangiogenesis, with significant correlations identified between M2 TAMs’ density and tumor LVD in dozens of cancers.^11^ Correspondingly, the aggregation of CD4 + T cells which expressed RAMP1, a new target of lymphangiogenesis that can lead to a reduction of VEGF‐C and VEGF‐D when it knocked down, in inflammatory environments is also positively correlated with lymphangiogenesis. These T‐cell types could be the sources of lymphangiogenesis cytokines.^52^

**FIGURE 1:**
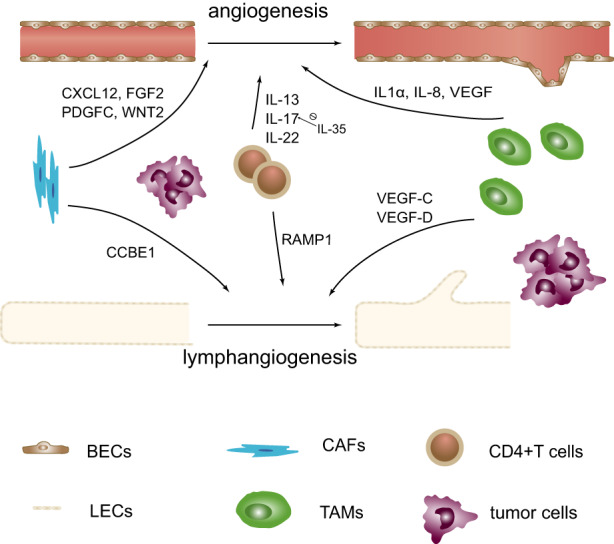
Molecular mechanisms of angiogenesis and lymphangiogenesis in tumors. Blood vessels and lymphatic vessels branch under the action of different factors secreted by various cells.

Since LECs are closely linked to CAFs, it was proved that PDGF‐D expressed by CAFs can stimulate human fibroblasts to secrete VEGF‐A and VEGF‐C. Furthermore, CAF depletion with drugs reduced lymphatic vascularization and lymphatic spread.^53^ CAFs also express and secrete collagen and calcium‐binding EGF domain‐1 (CCBE1) and promote VEGFC maturation and tumor lymphangiogenesis in colon cancer^54^ (Figure 1). In oral squamous cell carcinoma (OSCC), hepatocyte growth factor (HGF) was expressed in CAFs and regulated lymphangiogenesis by binding to the c‐Met receptor in OSCC.^55^

### 3.3 Membrane tube formation and its influencing factors

TNTs are membrane‐binding channels and are formed as follows: (i). A cell produces protrusions extending to the target cell, (ii). Both cells produce protrusions and connect, (iii). When both cells are adjacent, TNTs are formed when they separate.^56^ Recent research on TNT structure formation shows that two filopodia first protrude from two cells, respectively. They fuse through N‐cadherin molecules to become a double filopodial bridge when they contact each other, which could ward off their retraction. Subsequently, the double filopodial bridge continues to extend until they reach the paired cell body. At this time, N‐cadherin bonds destructed with the acquirement of torsional energy given by myosin V and myosin II. The double filopodial bridge attains the splitting moment that one filopodium turns to be TNT, whereas the other retracts back. N‐cadherin, as a mediator, fastens the TNT at the joint.^57^ Significantly, TNT formation is not only limited to individual cells, local networks between cell populations have been identified.^5^ Multiple cell types and environments would make a difference in the formation of these networks.

**FIGURE 2:**
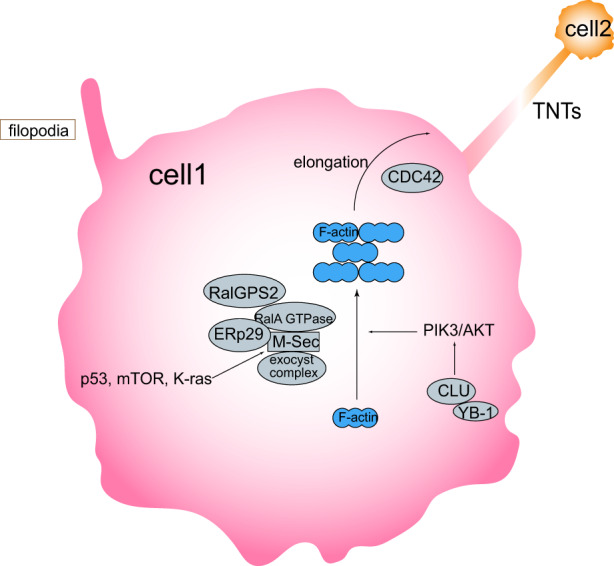
Molecular mechanism diagram of TNT formation. Cell 1 shows the molecules and pathways that are known to be involved in the formation of TNTs, initially forming filopodia, and connected to cell 2.

TNTs have long been described as adaptive response mechanisms to cellular stress. Multiple stress stimulation pathways increase TNT formation when cancer cells require increased metabolic input or are faced with hypoxia pressure and require preferential communication between stressed and non‐stressed cells. During stress, the stress chaperones, CLU, and YB‐1 impact TNT formation via the phosphatidylinositol‐3‐kinase (PI3K) pathway,^58^ and other conditions including hyperglycemic, low‐serum, acidic environments^16^ (Figure 2). Regardless of the environment, TNT formation requires the participation of a variety of regulatory proteins. The earliest discovered marker was M‐Sec, a tumor necrosis factor‐alpha‐induced cytosolic protein, which induced actin polymerization.^59^ M‐Sec is found in the signaling pathways such as p53, mTOR, and K‐RAS^60^ and participates in actin remodeling and polymerization by binding to the RalA small GTPase and exocyst complex, and requires the endoplasmic reticulum chaperone, ERp29 for stabilization.^61^ During this process, a Ras‐independent guanine exchange factor with PH domain and SH3‐binding motif 2 (RalGPS2) was demonstrated to promote RalA to interact with Sec5 and leukocyte‐specific transcript 1, playing a critical role in nanotubes generation, a new molecule involved in the multimolecular complex formation.^62^ If one of two cells does not express M‐sec, filopodial bridges with blind ends will be formed.^58^ Subsequently, the small GTPases induce actin polymer elongation, during which, the Rab11a‐Rab8a cascade regulates TNT formation.^63^ In addition, TNTs elongation also requires CDC42 participation.^59^

Currently, little is known about the formation mechanism behind TMs; however, GAP43 was identified as a marker and the main driving factor of TMs. And, it appears that although TMs contain actin, connexin 43 is the main structural component.^6^, ^18^ To better understand the determinants affecting TM formation, a new functional methodology was adhibited. By isolating TM‐connected cells and TM‐unconnected cells, Nestin, a marker of cellular stemness, has recently been identified as a core protein present in the glioma TM networks in vivo.^64^ Through this study, Joseph et al. identified TMs with nestin and finally demonstrated that TGFB1‐TSP1 is the main induction pathway with SMAD3 as the main TSP1 transcription factor, which jointly affect TM formation.^65^

## 4 NEOGENESIS PIPELINE FUNCTIONS AND ITS ROLE IN TUMOR PROGRESSION

### 4.1 Neoplastic angiogenesis ‐ functions and carcinogenic role

Due to angiogenesis, tumors acquire nutrients and oxygen for development. Newly generated vascular networks also remove metabolic waste and carbon dioxide from TME, promote metabolic regulation,^66^ and enhance tumor stem cell persistence.^67^ Neovascularization is a key tumor metastasis component and was proposed by Folkman in 1971.^68^ This process arises from immature blood vessel structures, and vascular permeability factor/VEGF contributes to vessel permeability,^69^ leading to tumor cell infiltration. Similarly, new blood vessels also lead to local tumor infiltration, increasing tumor cell movement and invasion.^70^ In addition, angiogenesis, as an important predictor of tumor behavior, is used to identify patients with recurrent disease and early death predictions. These predictors are also helpful for postoperative staging.^71^

In recent years, the generation of new blood supplies due to VM has attracted much research attention. Some tumor stem cells can change their original function, imitating endothelial cells and allowing red blood cells to flow in blood vessels and continue to provide blood for malignant tumors.^72^ Not only are VM‐mediated tumors more likely to metastasize,^4^ VM formation after tumor surgery is cited in treatment failure.^73^ Critically, VMs are also significantly correlated with the poor survival of patients.^74^

### 4.2 Neoplastic lymphatic ‐ functions and carcinogenic role

Lymphangiogenesis is a key initial step in lymphatic and regional lymph node metastasis.^75^ Lymphatic vessels are the main transmission route for a variety of malignant tumors. Similar to the way blood vessels promote tumor metastasis, tumor LECs facilitate metastasis caused by their high permeability of monolayer, which is convenient for tumor cell invasion.^50^, ^53^ Tumor LVD has a positive correlation with intravascular infiltration, lymph node metastasis, tumor recurrence, and reduced patient survival. In metastatic melanoma, it had more intratumoral lymphatic vessels than non‐metastatic melanoma, and lymphatic vessel areas were related to disease‐free and overall survival.^76^ The study indicated that higher LVD could lead to a higher risk of lymph node metastasis. Conversely, it was identified that in colon cancer, LVD in the invasive margin of the primary tumors has a significant decrease in patients with metastasis tumors.^13^ The same conclusion is also demonstrated in prostate cancer.^77^ These two opposing results suggest that lymphangiogenesis and tumor metastasis may be tissue specific.

The function of lymphangiogenesis is twofold during immune responses or immunotherapy. The process helps tumor cells escape immune responses, where VEGFC/VEGFR3 pathway plays a role. In a mouse colon cancer model receiving VEGF‐C, CD3+ cells were reduced and more T lymphocytes were identified after blocking VEGF‐C. The VEGFC/VEGFR3 pathway affects tumor immunity by inhibiting T‐cell proliferation. Not only that, but the pathway also polarizes macrophages into immunosuppressive cells.^78^ However, lymphangiogenesis is also very important during adaptive immune responses, as it transports immune cells and antigens, especially in some tumors lacking lymph node drainage, such as brain tumors. Ectopic VEGF‐C expression activates CD8 T cells in deep cervical lymph nodes and contributes to their migration to glioblastoma, revealing their ability to promote tumor immunodetection.^79^ Based on this, Maria Stella Sasso et al. used lethally irradiated tumor cells, overexpressing VEGF‐C to create a lymphangiogenic vaccine which drove the immune response system in mouse melanoma models and identified new prospects for antitumor vaccines.^80^

### 4.3 Tumor membrane tubes ‐ functions and carcinogenic role

#### 4.3.1 TNT functions and carcinogenic role

TNTs promote tumor invasion by inducing phenotypic changes in target cells by transporting hereditary materials, like microRNAs. For example, in bladder cancer, miR‐155 in TNTs was transported from highly aggressive to less aggressive cells and activated bladder cancer cell reprogramming via mTOR signaling.^15^ Increased cell communications mediated by TNTs link wild‐type and KRAS‐mutated cancer cells to induce phenotypic changes in colorectal cancer cells were also demonstrated.^81^ Furthermore, TNTs transfer miRNAs from cancer cells to endothelial cells; TNTs allow these latter cells to acquire aggressive molecules,^82^ such as miR‐132, which were not expressed in these cells. Studies have also indicated that cancer cells can form nanoscale ducts with endothelial cells and contribute to their metastatic potential.^83^ TNT formation between tumor cells and macrophages generates more aggressive tumor phenotypes, which can be detected according to tumor cell elongation, for tumor cell elongation increased by 60% in the presence of macrophages. M‐sec deletion from macrophages, or TNT‐defective macrophages, cannot induce tumor invasion into the collagen layer when compared with controls.^84^ In gliomas, tumor‐derived mitochondria are transferred into non‐tumor astrocytes, resulting in tumor‐related metabolism to elicit astrocytes to adapt to hypoxic conditions. Thus, TNTs, as critical mediators, could persist in cancer stem‐like cells' survival by changing their phenotypes to adapt to TME when they are localized at the edge of the tumor.^85^

In addition to the above function, TNTs also promote angiogenesis using time‐lapse imaging to observe TNTs formed between the tumor and human vascular endothelial cells and find the transmission of VEGF and HIP‐1.^73^ Substantial studies reported that TNTs from pericytes may have roles in the early stage of tumor blood vessels and participate in pathological tumor angiogenesis by connecting pericytes or connecting pericytes to endothelial cells during glioblastoma.^86^

TNTs can generate chemotherapy and radiation resistance between different tumor cells, for example, TNTs induced glioblastoma resistance in sensitive cells and prevented apoptosis during ionizing radiation and temozolomide treatment.^87^ The same phenomenon was observed in pancreatic cancer cell lines and ovarian cancer cell lines; after these two cell lines were exposed to doxorubicin, TNTs were formed in a dose‐dependent manner, with doxorubicin successfully transferred from doxorubicin‐positive cells to recipient cells via TNTs, with apoptosis occurring in chemotherapy‐sensitive cells.^8^ Further, TNTs participated in classic multidrug breast cancer resistance patterns by delivering p‐glycoprotein to increase breast cancer cell resistance.^88^ Regardless of chemotherapy‐sensitive receptor cell death, TNTs mediated drug outflow or redistribution between cells, allowing these cell networks to protect themselves, thereby increasing the survival of drug‐resistant cells. This redistributive property limited treatment accuracy and specificity by forming isolated tumor niches.^89^ Antitumor drugs can also be transported by TNTs between tumor cells and macrophages.^90^ In addition, given that one of the effects of cytotoxic drugs on tumor cells is to initiate free radicals in mitochondria, myeloma cells can be associated with bone marrow stromal cells to transfer distressed mitochondria to bone marrow stromal cells. And get new mitochondria from there so that the number of their free radicals is significantly attenuated.^91^

#### 4.3.2 TM functions and carcinogenic role

The key function of the TM cellular network is the bidirectional transport of calcium between cells. In gliomas, even a small increase in intracellular calcium levels can lead to glioma cell death because of an increased intracellular calcium level required for radiotherapy‐induced cytotoxicity.^92^ Intercellular TMs allow individual tumor cells to distribute critical, small molecules such as calcium to larger networks, reaching non‐lethal levels, thus, increased TMs during radiotherapy are viewed as a radiotherapy response.^18^ Herein, this characteristic is due to NOTCH1 signaling pathway regulation. When NOTCH1 is downregulated, TM‐connected multicellular networks are formed to compensate for the depletion of tumor cells in the perivascular niche.^93^ Most malignant astrocytomas recur after treatment and possibly arise due to the fact that tumor cells stretch TMs to surgically damaged sites, proactively repair injuries, and eventually form new tumor masses at the edge of surgical resection, indicating that TMs are also involved in the reaggregation process.^6^

## 5 POTENTIAL APPLICATIONS OF TUMOR REGENERATION PIPELINES FOR TARGETED TUMOR THERAPIES

With an increased understanding of disease mechanisms and continuous technical advancements, targeted tumor therapies have become progressively important, with tumor target and mechanism research escalating in recent years. At present, research based on tumor regeneration pipelines has become a vital research area for the identification of new therapeutic strategies (Table 1).

**TABLE 1:** Antitumor drugs targeting tumor neogenesis pipeline

Antitumor drugs	Tumor types	Target	Effect	Ref
Sorafenib	Triple‐negative breast cancer orthotopic glioblastoma	Inhibitors of VEGF receptor tyrosine kinase activity	Increased tumor invasion	72, 96
Sunitinib	Triple‐negative breast cancer	Inhibitors of VEGF receptor tyrosine kinase activity	Hypoxia increased the expression of Twist and induced VM formation	^98^
Dequalinium‐modified paclitaxel plus ligustrazine micelles	Non‐small‐cell lung cancer	Inhibitors of VMs	Inhibit tumor metastasis	^99^
Niclosamide	Oral cancer	Upregulation of miR‐124 and downregulation of STAT3, inhibit VMs formation	Decrease the size of tumor and the number of VMs in mouse models	^100^
Dual anti‐PD‐1/VEGFR‐2 therapy	Hepatocellular carcinoma	Change the TME, reduce Treg and part of monocyte infiltration, change the proportion of M1/M2 macrophages, and promote vascular normalization	Overcome resistance to drug therapy alone and improve overall survival	^106^
Combined ANGPT2 and VEGFA blockade by A2V	Breast cancer, pancreatic neuroendocrine tumors, colorectal adenocarcinomas	Promote vascular atrophy, tumor necrosis, vascular normalization and promote activation of CD8+ T lymphocytes, and interferon‐γ exudation	Increased the survival time of transgenic tumor mice with almost no metastasis	^71^
Navitoclax	Cholangiocarcinoma	Reduce lymphatic vascularization and lymph node metastasis	Reduce lymph node metastasis	^16^
GTPase inhibitors	Breast cancer	Reduce the invasiveness of tumor cells		^33^

### 5.1 Potential therapeutics targeting tumor neovascularization

**FIGURE 3:**
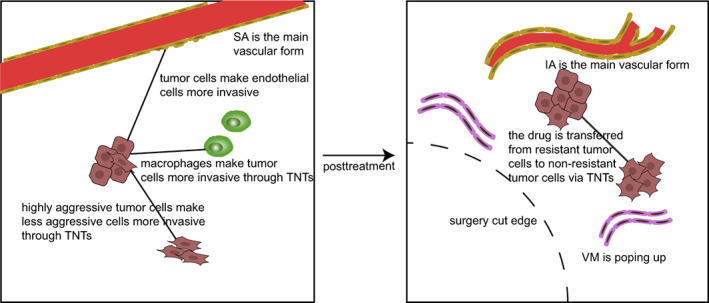
Changes in membrane tubes before and after clinical treatment. Before treatment, blood vessels were dominated by SA, and TNTs transported substances to make tumor cells more invasive. After treatment, the blood vessels were dominated by IA and a large number of VMs appeared. TNTs began to transport drugs for drug redistribution.

Anti‐angiogenic therapies (AATs) are used against multiple cancers; they mainly inhibit endothelial cell proliferation or induce endothelial cell death with a view to reducing blood vessel density. However, the clinical effects of these drugs are limited, partly due to the expression of alternative angiogenic pathways. As mentioned above, IA mostly appears after SA, especially during treatment with chemotherapeutic drugs. In animal studies, expansion of the capillary plexus was mainly observed in IA, with SA essentially replaced.^94^ This transformation was likely affected by nitric oxide (NO) production in tumor cells because NO could enhance angiogenesis and improve poor TME. In addition, NO donor administrated with Hela cells leading to a significant increase in IA‐like markers mRNA.^95^ This feature makes IA a new and potentially important therapeutic target. The efficacy of anti‐VEGF drugs toward triple‐negative breast cancer patients is limited, and the hypoxia induced by this VEGF inhibitor can promote tumor invasion and metastasis in mice, which is believed to be caused by angiogenesis,^96^ and this new vessel was proved to be VM.^73^ Additionally, several studies reported that tumor cells with VM, EMT regulatory factors, and related transcription factors were highly upregulated as they promoted VM formation and significantly enhanced tumor invasion.^97^ Hypoxia caused by some drugs accelerated VM production by cancer stem cells.^98^ The focus of the study turned to effective drugs to inhibit VM, which could lower the expression of VM‐related genes.^99^, ^100^ Thus, these inhibitors are potential drug candidates for anti‐VM therapies and suggest that drug combinations targeting angiogenesis and VM can inhibit tumor growth more effectively than single drugs^101^ (Figure 3).

In order to break out of this cycle, that is, AATs promoting tumor angiogenesis, vascular normalization could correct tumor blood vessels immaturity and be an emerging therapeutic strategy. The so‐called normalization of blood vessels is the process of returning abnormal blood vessels to normal blood vessels.^102^ Vascular normalization destruction reduces T lymphocyte infiltration, whereas the mutual depletion or inactivation of CD4+ T lymphocytes reduces VM. Lin Tian et al. demonstrated that Type 1 T helper (Th1) cells were the main cell type in vascular normalization. Immune checkpoint blockers could block CTLA‐4 and PD‐1 in mice. Activation of CD4 + T cells promoted tumor vessel normalization.^103^ In addition, studies reported that during anti‐PD‐1 therapy, CD4+ mediated blood vessel normalization.^104^ Other antiangiogenic drugs can also directly or indirectly reduce the number of immunosuppressive cells in the TME and normalize tumor blood vessels.^104^, ^105^ Similarly, it was reported that a bispecific antibody (A2V) normalized blood vessels, increased T‐cell numbers, and upregulated programmed cell death ligand 1 (PD‐L1) expression at immune checkpoints, raising the possibility of improved anti‐vascular therapeutic effects when combined with anti‐PD‐L1 drugs.^106^

### 5.2 Potential therapeutics targeting tumor lymphangiogenesis

In comparison, few studies have been published on anti‐lymphangiogenesis. In 2010, Zhuo et al. used endostatin to target nucleolin expressed on the surface of endothelial cells that produce lymphatic vessels. In a mouse melanoma model treated with nucleolin, lymph node metastasis was significantly reduced, indicating the nucleolin‐mediated antitumor lymphogenesis functions of endostatin.^107^ Further, parts of angiogenesis inhibitors are corroborated to inhibit lymphangiogenesis. The abovementioned VEGF‐A antibody neutralization or anti‐VEGF‐A therapy was shown to inhibit lymphangiogenesis and lymphatic metastasis during breast cancer.^48^ Presently, it was suggested that CAFs could also be a potential target, with a reduction in lymphatic vascularization and lymph node metastasis observed after CAFs elimination of the rat model of cholangiocarcinoma treated with Navitoclax.^53^ The characteristic of lymphangiogenesis in CAFs also demonstrated in renal cell carcinoma indicated that targeting CAFs will become a powerful therapy in multiple solid tumors.^108^

### 5.3 Potential therapeutics targeting tumor membrane tubes

In solid tumors, TNTs may be targeted by novel targeted therapies. Such therapeutic drugs are divided into two classes. The first acts on TNT generation. Some therapeutics inhibit TNTs before formation, that is, inhibit filopodia. Nickolay et al. showed that CytoB specifically removed TNT filopodia precursors in PC12 cells, which substantially reduced organelle exchange.^109^ Similarly, other conventional drugs target TNT growth and inhibit the actin tissue‐related signaling pathway, mTOR, implicated in TNT formation. Metformin exerted significant effects on TNTs in mesothelioma cells and inhibited glycolytic migration by suppressing gluconeogenesis in glioma cells. Apparently, the mTOR pathway became overactivated under hyperglycemic conditions, which means metformin can indirectly affects this pathway.^16^ By using small Rho GTPases inhibitors, which inhibit the actin polymerization step, when co‐cultured with endothelial cells, it reversed more aggressive cancer cells into smaller aggressive mammosphere‐like structures.^82^ The second class exploits the transfer properties of TNTs. Deng et al. observed nanoparticles transfer between HeLa cells in an experiment using silicon dioxide as a carrier and hypothesized such transfers were associated with TNTs.^110^ TNTs may serve as potential drug delivery channels, for example, nanomaterials were transported in TNTs using fluorescent nanodiamonds as vesicle carriers to facilitate cell‐to‐cell communications.^111^ Studies have shown that when compared with mApoE and chlorotoxin peptide (MF‐LIP) exchange between normal astrocytes, U87‐MG cells were more efficient in transmitting MF‐LIP between them, which is due to more LIP. The localization is in the thick TNTs, while the U87‐MG cell line just forms more thick TNTs. This phenomenon allows drugs to effectively reach isolated tumor cells.^89^ In gliomas, where drugs have a hard time crossing the blood–brain barrier, microglia can deliver freely. Engineered microglia loaded with paclitaxel were generated. These engineered microglia establish intercellular communication specifically to glioma cells and deliver drugs through TNTs.^112^

These emerging therapeutic strategies have greatly improved targeting accuracy and maximized the therapeutic drug effects. Recently, it was reported that TNTs functioned as transportation expressways between tumor cells and macrophages to significantly increase doxorubicin delivery efficiency to ovarian cancer cells, which is much faster than free doxorubicin delivery and this efficiency was reduced when M‐Sec small interfering RNA inhibited TNT formation.^90^

## 6 CONCLUSIONS AND PROSPECTIVES

Unequivocally, tumor pipeline research and data outputs have increased in recent years. It is clear that blood vessels, lymphatic vessels, or membrane tubes increase the potential for malignant tumor development. Blood and lymphatic vessels are closely related and physically connected. Tumor cells reach the blood circulation directly via capillaries or indirectly via lymphatic vessels. Almost all inflammatory cells that promote angiogenesis will promote lymphangiogenesis, thus, both mechanisms complement each other, and contribute considerably to the survival and migration of malignant tumors. TNTs and TMs, as means of communication between cells, have different structures and exist in discrepant tumor types. In terms of cargo transportation, they are also quite different. From a clinical perspective, these structures exert similar cancer‐promoting and tumor invasion effects. Additionally, blood and lymphatic vessel formation may lead to postoperative tumor recurrence, and similarly, these membrane tubes may induce tumor drug resistance. By understanding the formation mechanisms underpinning these conduits, novel clinical diagnostics and treatment strategies can be designed.

In the future, several targeted therapy factors must be considered for tumor neoplastic pipelines. Firstly, the combination of anti‐VM drugs or antibodies that normalize blood vessels should be considered in anti‐vascular therapy to improve chemotherapy efficiency. Secondly, more experiments are required to investigate anti‐lymphangiogenesis treatments, as this process is closely associated with tumor metastasis. Thirdly, emerging nanotechnologies could be exploited to transport drugs to tumor cells with the help of TNTs. Exploring the specific mechanisms of tumor pipeline generation, how they promote tumor cell invasion, and how tumor cells develop drug resistance, will provide powerful tools for clinical treatment. Thus, blocking tumor pipeline formation has the potential to reduce drug resistance and recurrence rates by pharmacologically targeting tumor networks. Exploring these questions will provide key insights for cancer treatment.

## AUTHOR CONTRIBUTIONS

YY conceived the study. ZQ‐F systematically collected literature and drafted the manuscript. YY revised the full text. All authors read and approved the final manuscript.

## FUNDING INFORMATION

This study was supported by The National Key R&D Program of China (2018YFC1311600)

## Data Availability

Data sharing is not applicable to this article as no new data were created or analyzed in this study.
